# Effects of diabetes mellitus on left ventricular function and deformation in patients with restrictive cardiomyopathies: a 3.0T CMR feature tracking study

**DOI:** 10.1186/s12933-023-02033-w

**Published:** 2023-11-20

**Authors:** Yue Gao, Yi-Ning Jiang, Rui Shi, Ying-Kun Guo, Hua-Yan Xu, Chen-Yan Min, Zhi-Gang Yang, Yuan Li

**Affiliations:** 1 Department of Radiology, West China Hospital, Sichuan University, 37# Guo Xue Xiang, Chengdu, 610041 Sichuan China; 2 Department of Radiology, Key Laboratory of Birth Defects and Related Diseases of Women and Children of Ministry of Education, West China Second University Hospital, Sichuan University, Chengdu, Sichuan China

**Keywords:** Diabetes mellitus, Restrictive cardiomyopathy, Global peak strain, Cardiac magnetic resonance

## Abstract

**Background:**

Diabetes mellitus (DM) is the most common metabolic disease worldwide and a major risk factor for adverse cardiovascular events, while the additive effects of DM on left ventricular (LV) deformation in the restrictive cardiomyopathy (RCM) cohort remain unclear. Accordingly, we aimed to investigate the additive effects of DM on LV deformation in patients with RCM.

**Materials and methods:**

One hundred thirty-six RCM patients without DM [RCM(DM−)], 46 with DM [RCM (DM+)], and 66 age- and sex-matched control subjects who underwent cardiac magnetic resonance (CMR) scanning were included. LV function, late gadolinium enhancement (LGE) type, and LV global peak strains (including radial, circumferential, and longitudinal directions) were measured. The determinant of reduced LV global myocardial strain for all RCM patients was assessed using multivariable linear regression analyses. The receiver operating characteristic curve (ROC) was performed to illustrate the relationship between DM and decreased LV deformation.

**Results:**

Compared with the control group, RCM (DM−) and RCM(DM+) patients presented increased LV end-diastolic index and end-systolic volume index and decreased LV ejection fraction. LV GPS in all three directions and longitudinal PDSR progressively declined from the control group to the RCM(DM−) group to the RCM(DM+) group (all p < 0.05). DM was an independent determinant of impaired LV GPS in the radial, circumferential, and longitudinal directions and longitudinal PDSR (β =  − 0.217, 0.176, 0.253, and − 0.263, all p < 0.05) in RCM patients. The multiparameter combination, including DM, showed an AUC of 0.81(95% CI 0.75–0.87) to predict decreased LV GLPS and an AUC of 0.69 (95% CI 0.62–0.76) to predict decreased LV longitudinal PDSR.

**Conclusions:**

DM may have an additive deleterious effect on LV dysfunction in patients with RCM, especially diastolic dysfunction in RCM patients, indicating the importance of early identification and initiation of treatment of DM in patients with RCM.

## Introduction

Diabetes mellitus (DM) is considered a major risk factor for cardiovascular complications and is an independent risk factor for cardiovascular morbidity and mortality [1, 2]. Left ventricular (LV) hypertrophy, myocardial fibrosis, stiffness, and diastolic dysfunction are the main courses of diabetic cardiomyopathy [3–5]. Restrictive cardiomyopathy (RCM) is a myocardial disorder with common physiology but divergent etiologies [6]. RCM patients have a stiff LV with impaired diastolic filling and high filling pressures, which commonly induce pulmonary hypertension and tend to exacerbate heart failure (HF). Until later stages of the disease, biventricular chamber size and systolic function are usually normal or almost normal [7–9]. However, the additive effects of DM on left ventricular (LV) deformation in the RCM cohort remain unclear.

Previous studies have pointed out that RCM and DM can impair LV function and deformation, culminating in progressive deterioration and poor outcomes [10, 11]. Similar to RCM, DM status can aggravate cardiac structural and functional abnormalities, such as diastolic dysfunction and LV wall stiffness [12, 13]. Therefore, among RCM patients, investigating the effects of DM on LV myocardial deformation is important to achieve the goal of health management.

Cardiac magnetic resonance (CMR) imaging provides comprehensive information on cardiac function, deformation, and myocardial tissue. The late gadolinium enhancement (LGE) can potentially enhance the diagnosis, management, and prognosis of RCM [14]. The deformation, especially impaired global longitudinal strain, has been proven to be associated with cardiovascular events and have a better prognostic value than LVEF [15, 16]. Therefore, the current study sought to investigate the additive effects of DM on LV function and global deformation in patients with RCM.

## Methods and materials

### Study population

The study protocol was approved by the Biomedical Research Ethics Committee of our hospital. Informed consent was waived due to the retrospective nature of the research.

Initially, we consecutively retrospectively enrolled 253 patients with RCM who had completed CMR examinations in our hospital between January 2010 and December 2022. The diagnostic criteria for RCM were invasive cardiomyopathy confirmed by biopsy or a combination of clinical symptoms and relevant cardiac examinations [17]. The exclusion criteria were as follows: (1) congenital heart disease, pericardial disease, severe arrhythmia, severe valvular heart disease, or acute coronary syndrome; (2) an incomplete clinical record; and (3) inadequate images because of arrhythmia or poor image quality. Following these criteria, a total of 182 RCM patients were included in this study. According to whether there was coexisting DM, patients were further divided into the RCM without DM (RCM[DM−]) group and the RCM with DM (RCM[DM+]) group. The diagnosis of DM was based on current European Society of Cardiology (2019) guidelines [18]. In addition, age-, sex-, and body mass index-matched subjects without a diagnosis of the RCM and a history of DM were enrolled as controls. We excluded patients with congenital heart disease, primary cardiac myopathy, pericardial disease, severe arrhythmia, severe valvular heart disease, coronary artery disease, MI, acute coronary syndrome, and cardiac MRI images with poor quality.

### CMR scanning protocol

All CMR examinations were performed in the supine position using a 3.0T whole body magnetic resonance scanner Trio Tim or MAGNETOM Skyra (Siemens Medical Solutions, Erlangen, Germany) equipped with 32-channel body phased array coils and a standard ECG trigger equipment. Balanced steady-state free precession (b-SSFP) cine images were acquired using a retrospective vector ECG gating technique at the end of inspiratory breath holding, and twenty-five frames were reconstructed per breath-hold acquisition. Standard short-axis, long-axis two- and four-chamber cine images were obtained. which covered the entire left ventricles. The following scanning parameters were used: repetition time (TR) 2.81 ms or 3.4 ms, echo time (TE) 1.22 ms, flip angle 40° or 50°, slice thickness 8 mm, field of view (FOV) 250 × 300 mm^2^ or 340 × 285mm^2^, and matrix 208 × 139 or 256 × 166. Gadolinium-based contrast agent (MultiHance; Bracco, Milan, Italy; Magnevist, Bayer Schering Pharma, Berlin, Germany) was intravenously injected at a dose of 0.2 mmol/kg body weight at an injection rate of 2.5–3.0 ml/s, followed by a 20 ml saline flush at a rate of 3.0 ml/s. LGE images were acquired in the corresponding slice position as the cine imaging 10–15 min after contrast injection. The images were obtained using a phase-sensitive inversion recovery sequence with the following parameters: temporal time 300 ms, TE 1.44 ms, flip angle 40°, slice thickness 8 mm, FOV 275 × 400 mm^2^, and matrix size = 256 × 184 mm^2^.

### CMR data analysis

All CMR data were uploaded to an offline workstation using a semi-automated software (Cvi42; Circle Cardiovascular Imaging, Inc., Calgary, Canada). The LV endocardial and epicardial traces were manually or semiautomatically delineated in the serial short-axis slices at the end-diastolic and end-systolic phases. Papillary muscles were considered as part of the ventricular cavity, and epicardial fat was excluded. LV functional parameters, including LV end-diastolic volume (LVEDV), LV end-systolic volume (LVESV), LV stroke volume (LVSV), LVEF, and LV mass (LVM) were computed automatically. LVEDV, LVESV, LVSV and LVM were indexed to body surface area (BSA). The LV global function index (LVGFI) was calculated using the following formula [19]:

$${\text{LVGFI}}=\{{\text{LVSV}}/[({\text{LVEDV}}+{\text{LVESV}})/2+({\text{LVM}}/1.05)]\}\times 100$$

**Fig. 1:**
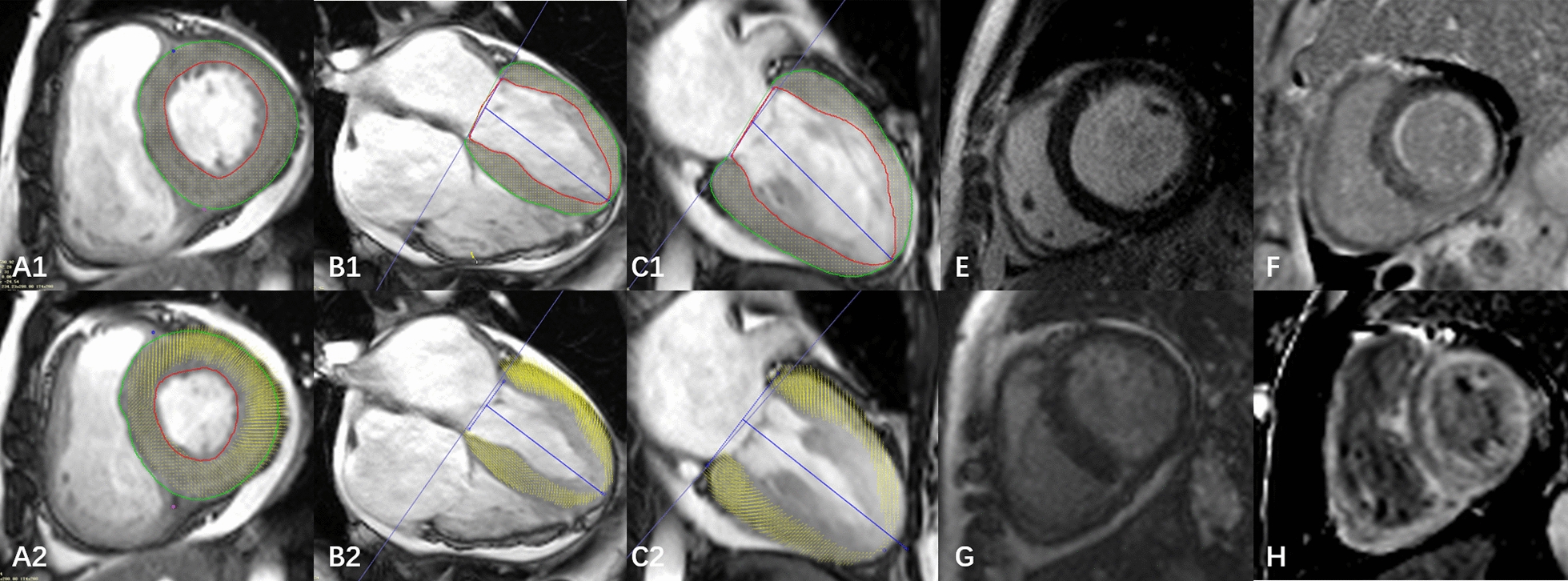
Measurement of LV global strain and definition of LGE patterns. Cardiac magnetic resonance feature tracking in short‑axis and long‑axis four‑chamber and two‑chamber cine images at end‑diastole (**A1**, **B1**, **C1**) and end‑systole (**A2**, **B2**, **C2**). LGE patterns were defined as none (**E**), focal patchy (**F**, **G**) and global (**H**)

For LV global deformation analysis, LV long-axis cine images (2-chamber and 4-chamber) and short-axis cine (2-chamber) images were loaded into the feature tracking module by delineating LV endocardial and epicardial borders at the end-diastolic phases of all cine images. The LV global radial peak strain (GRPS), global circumferential peak strain (GCPS), and global longitudinal peak strain (GLPS) and their corresponding peak systolic strain rate (PSSR) and peak diastolic strain rate (PDSR) in the three directions were acquired automatically (Fig. 1). LEG was defined as the area of signal intensity five standard deviations above the mean intensity of the normal myocardium on the LGE short-axis images. Two radiologists (Y.G and R.S) categorized delayed enhancement into 1 of 3 categories: (1) None: in which there were no areas of LGE; (2) Focal patchy: in which there were non-diffuse, discrete areas of LGE, including circumferential LGE confined to the endocardium; (3) Global: in which there was circumferential, diffuse LGE extending from the endocardium to the epicardium (Fig. 1) [20]. These two observers evaluated the LGE images separately, and if the results were inconsistent, they discussed and agreed on the result.

### Reproducibility analysis of LV strain

To determine intra- and inter-observer variability, one observer (Y. G) measured LV global myocardial strain and strain rate in 60 random subjects (including 40 RCM patients and 20 controls) twice within one month. A second observer (R. S), who was blinded to the results of the first observer and clinical data, reperformed the measurements to assess the interobserver variability.

### Statistical analysis

Statistical analyses were performed with SPSS (version 23.0; IBM SPSS, Inc., Chicago, IL, USA). Data are expressed as the means with standard deviations or medians with interquartile ranges (IQRs) for continuous variables. Categorical variables are presented as numbers (percentages) and compared using Fisher’s exact test or the chi-square test, as appropriate. Parameters among RCM(DM−), RCM(DM+), and control were compared by one-way analysis of variance (ANOVA) followed by Bonferroni’s post hoc test (normally distributed variables) or the Kruskal–Wallis rank test (nonparametric variables), as appropriate. Correlation analysis was conducted to identify the relationship between LV function, strain parameters, and clinical indexes. Pearson’s correlation was used between continuous variables, and Spearman’s correlation was used to analyze the rank correlation.

Moreover, variables with a p-value of less than 0.1 in the univariable analyses and an absence of collinearity were included in a stepwise multivariable analysis to identify the independent determinants of LV strain parameters. The receiver operating characteristic curve (ROC) was performed to quantify the diagnostic efficiency of DM for LV impaired deformation. A p-value of < 0.05 was considered statistically significant.

## Results

### Baseline characteristics

Overall, 182 RCM (RCM[DM−]: n = 136, 61.03% male, 58.35 ± 10.76 years; RCM[DM+]: n = 46, 58.70% male, 58.46 ± 11.97 years) patients and 66 controls (68.18% male, 57.42 ± 8.65 years)were included in this study. The main clinical baseline characteristics of the study cohort are summarized in Table 1. Age, sex, BMI, systolic blood pressure, diastolic blood pressure, and heart rate were not significantly different between the observed groups (all p > 0.05). Cardiac amyloidosis is the predominant type of RCM, whether or not these patients have DM. Regarding cardiovascular risk factors, there was no difference in hyperlipidemia, hypertension, or atrial fibrillation between the two RCM groups (all p > 0.05). The NYHA functional class in the RCM(DM+) group was decreased than in the RCM(DM-) group (p < 0.05). Additionally, the NT-proBNP value was significantly higher in the RCM (DM+) group than in the RCM (DM−) group (7642.00[1337.00–13602.00] vs. 4322.50[1436.00–7777.75], p < 0.05), and there was no difference in troponin, eGFR or creatinine values between the two RCM groups. For the antidiabetic medication of RCM(DM +) patients, 11 patients used insulin, and 40 patients used oral antidiabetic agents (22 for biguanides, 5 for sulfonylureas, 15 for α-Glucosidase inhibitor, and 3 for GLP-1/DPP-4 inhibitor).

**Table 1:** Baseline characteristics of the study cohort

	Control subjects (n = 66)	RCM(DM−) (n = 136)	RCM (DM+) (n = 46)
Baseline characteristics			
Age, years	57.42 ± 8.65	58.35 ± 10.76	58.46 ± 11.97
Male, n (%)	45(68.18%)	83(61.03%)	27(58.70%)
BMI, kg/m^2^	23.31(21.78,25.27)	22.15 ± 2.95	22.04 ± 3.00
Systolic BP, mmHg	127.83 ± 16.19	100.84 ± 22.41	110.26 ± 26.51
Diastolic BP, mmHg	76.58 ± 10.16	77.50(68.00–90.00)	80.50(71.00–90.50)
Heart rate, bpm	73.74(65.25,78.97)	85.54(76.73,95.38)^*^	91.44(80.26,103.03)^*^
Cardiac amyloidosis, n (%)		114(83.8%)	42(91.3%)
Cardiac risk factors, n (%)			
Hypertension	–	40(29.41%)	25(54.35%)^§^
Hyperlipidemia	–	11(8.09%)	5(10.87%)
Atrial fibrillation	–	15(11.0%)	4(8.69%)
NYHA functional class, n			
I/II/III/IV	–	6(4.41%)/51(37.50%)/58(42.65%)/21(15.44%)	1(2.17%)/6(13.04%)/27(58.70%)/12(26.09%)^§^
Laboratory data			
HbA1c, %	–	–	7.40(6.80,8.00)
eGFR, mL/min/1.73m^2^	–	79.94 ± 28.45	71.91 ± 33.67
Creatinine, umol/L	–	78.00(58.00,102.50)	81.00(69.50,126.25)
Troponin, ng/L	–	78.55(39.85,138.80)	92.40(45.25,149.15)
NT-proBNP	–	4322.50(1436.00–7777.75)	7642.00(1337.00–13602.00)^§^

*DM* diabetes mellitus, *RCM* restrictive cardiomyopathy, *BMI* body mass index, *BP* blood pressure, *NYHA* New York Heart Association, *HbA1c* glycated hemoglobin, *eGFR* estimated glomerular filtration rate

^*^p < 0.05 versus control group (Bonferroni’s)

^§^p < 0.05 versus RCM patients without DM

### Comparison of CMR parameters among RCM patients with and without DM and controls

The CMR imaging results for LV function and global peak strain were summarized in Table 2. In contrast to the controls, patients who had RCM with and without DM exhibited increased LVEDVi, LVESVi, LVMI, and decreased LVSVi, LVEF, and LVGFI (all p < 0.05). The RCM (DM+) group exhibited a lower LVSVi, LVEF, and LVGFI than the RCM (DM−) group (all p < 0.05). Regarding LV global deformation parameters, the LV global peak train in all three directions were decreased from the control group to the RCM (DM−) group to the RCM (DM+) group (all p < 0.005). Moreover, The LV global PDSR in the longitudinal direction (PDSR_L) declined progressively from the control group to the RCM (DM−) group to the RCM (DM+) group (0.98[0.77–1.11] vs. 0.53[0.42–0.69] vs. 0.44[0.35–0.50], all p < 0.05). The remaining LV global PSSR and PDSR parameters were decreased in the two RCM groups than in the control group (p < 0.05), but there were no statistical differences between the two RCM groups. In addition, global diffuse was the most common LGE pattern (47.7% in the RCM [DM−] group; 45.6% in the RCM [DM+] group), followed by focal patchy (29.4% in the RCM [DM−] group; 23.9% in the RCM [DM+] group), but there was no statistical difference in the LGE pattern between the RCM patients with and without DM (p > 0.05).

**Table 2:** CMR findings between control, RCM (DM−) group and RCM (DM+) group

	Control subjects (n = 66)	RCM (DM−) (n = 136)	RCM (DM+) (n = 46)
LVEDVi, ml/m^2^	70.96(62.63,80.16)	76.93(67.08,88.48)^*^	76.26(65.96,84.89)^*^
LVESVi, ml/m^2^	23.96(19.91,28.49)	36.34(29.21,46.64)^*^	36.79(29.98,53.47)^*^
LVSVi, ml/m^2^	47.68(40.86,53.31)	38.70(30.41,48.22)^*^	32.20(24.81,42.04)^*,§^
LVEF, %	65.47(62.57,70.24)	51.28(42.39,60.98)^*^	44.63(32.19,59.37)^*,§^
LVMI, g/m^2^	40.80(35.95,46.35)	71.01(56.42,88.56)^*^	63.29(50.88,88.59)^*^
LVGFI	50.89(47.76,55.72)	31.22(23.29,40.89)^*^	26.07(20.37,35.71)^*,§^
LVMVR	0.57(0.50,0.66)	0.91(0.74,1.15)^*^	0.83(0.68,1.14)^*^
LV GPS, %			
Radial	36.37(32.67,41.48)	14.69(10.18,21.26)^*^	9.72(7.50,19.31)^*,§^
Circumferential	− 20.59(− 22.67,− 19.01)	− 11.45(− 15.69,− 9.02)^*^	− 10.35(− 14.61,− 7.06)^*,§^
Longitudinal	− 15.35(− 17.14,− 12.73)	− 6.43(− 8.19,− 4.49)^*^	− 4.93(− 6.89,− 2.92)^*,§^
LV PSSR (1/s)			
Radial	2.10(1.79,2.57)	1.04(0.67,1.59)^*^	0.95(0.68,1.59)^*^
Circumferential	− 1.02(− 1.16,− 0.93)	− 0.83(− 1.10,− 0.61)^*^	− 0.78(− 1.02,− 0.57)^*^
Longitudinal	− 0.79(− 0.93,− 0.69)	− 0.51(− 0.73,− 0.36)^*^	− 0.46(− 0.71,− 0.31)^*^
LV PDSR (1/s)			
Radial	− 2.79(− 3.20,− 2.24)	− 1.07(− 1.77,− 0.79)^*^	− 0.97(− 1.40,− 0.61)^*^
Circumferential	1.34(1.21,1.52)	0.84(0.65,1.09)^*^	0.85(0.59,1.04)^*^
Longitudinal	0.98(0.77,1.11)	0.53(0.42,0.69)^*^	0.44(0.35,0.50)^*,§^
LGE pattern, n			
None/focal patchy/global	–	31/40/65	14/11/21

Data are presented as median (25th, 75th percentile)

*LVEDVi* left ventricular end diastolic volume index, *LVESVi* left ventricular end systolic volume index, *LVSVi* left ventricular stroke volume index, *LVEF* left ventricular ejection fraction, *LVMI* left ventricular mass index, *LVGFI* left ventricular global function index, *RCM* restrictive cardiomyopathy, *GPS* global peak strain, *PSSR* peak systolic strain rate, *PDSR* peak diastolic strain rate, *LGE* late gadolinium enhancement

^*^p < 0.05 versus control group (Bonferroni’s)

^§^p < 0.05 versus RCM(DM-) group (Bonferroni’s)

### Association of LV dysfunction and remodeling with clinical variables in RCM patients

Univariable and multivariable linear regression analyses were performed to evaluate the independent effect of DM on LV function and deformation in RCM patients. After multivariable adjustment for covariates among all RCM patients, DM was an independent determinant of impaired LVEF (β = 0.166, p = 0.014). Furthermore, NT-proBNP levels were independently associated with LVEF, LVMI, and LVMVR (β = − 0.260, 0.314, and –0.272, all p < 0.05), LGE was independently associated with LVEF and LVMVR (β = − 0.297 and 0.158, all p < 0.05) type, gender were independently associated with LVMVR(β = − 0.182, p < 0.05)(Table3).

**Table 3:** Determinants of LV dysfunction in RCM patients

	LVEF				LVMI				LVMVR			
	Univariable		Multivariable		Univariable		Multivariable		Univariable		Multivariable	
	r	p	β	p	r	p	β	p				
Age, years	0.017	0.819			0.012	0.870			− 0.023	0.761		
Male, n (%)	− 0.034	0.649			− 0.113	0.128			− 0.155	0.037	− 0.182	0.010
BMI, kg/m^2^	0.124	0.094			− 0.064	0.393			− 0.051	0.492		
Hyperlipidemia	0.070	0.348			0.003	0.972			0.075	0.314		
Hypertension	− 0.099	0.185			0.064	0.390			0.008	0.920		
DM	− 0.150	0.043	− 0.166	0.014	− 0.071	0.228			− 0.069	0.356		
eGFR, mL/min/1.73m^2^	0.069	0.395			0.015	0.851			0.009	0.393		
NT-proBNP^a^	− 0.371	< 0.001	− 0.260	< 0.001	0.328	< 0.001	0.314	< 0.001	0.350	< 0.001	0.272	< 0.001
LGE type	0.399	< 0.001	− 0.297	< 0.001	0.238	< 0.001			0.287	< 0.001	0.158	0.037

*DM* diabetes mellitus, *RCM* restrictive cardiomyopathy, *BMI* body mass index, *BP* blood pressure, *NYHA* New York Heart Association, *HbA1c* glycated hemoglobin,; *eGFR* estimated glomerular filtration rate, *LVEDVi* left ventricular end diastolic volume index, *LVESVi* left ventricular end systolic volume index, *LVSVi* left ventricular stroke volume index, *LVEF* left ventricular ejection fraction, *LVMI* left ventricular mass index, *LVGFI* left ventricular global function index, *GPS* global peak strain, *PSSR* peak systolic strain rate, *PDSR* peak diastolic strain rate, *LGE* late gadolinium enhancement

^a^NT-proBNP was log-transformed before being included in the regression analysis

As shown in Tables 4 and 5, after adjusting for confounding factors, the multivariable linear regression analysis showed that DM was independently associated with LV GRPS (β = − 0.217, p < 0.001),GCPS (β = 0.176, p = 0.005), GLPS (β = 0.253, p < 0.001), and PSDR_L(β = − 0.263, p < 0.001). Moreover, NT-proBNP level, LGE type, and LVMI were independently associated with LV GRPS (β =  − 181, –0.379 and –0.269, all p < 0.01), GCPS (β = 0.299, 0.280 and 0.209, all p < 0.01), GLPS (β = 0.249 0.330 and 0.177, all p < 0.01), and PSDR_L (β = 0.196, 0.188 and 0.243, all p < 0.01).

**Table 4:** Univariable and multivariable linear regression analysis of LV global peak strain in RCM patients

	GRPS				GCPS				GLPS			
	Univariable		Multivariable		Univariable		Multivariable		Univariable		Multivariable	
	r	p value	β	p value	r	p value	β	p value	r	p value	β	p value
Age#, years	0.042	0.573			0.033	0.659			− 0.036	0.626		
Male, n (%)	0.057	0.446			− 0.092	0.216			− 0.008	0.918		
BMI, kg/m^2^	0.058	0.434			− 0.081	0.277			− 0.122	0.101		
NYHA	− 0.251	0.001			0.262	< 0.001			0.280	< 0.001		
Hyperlipidemia	0.044	0.554			− 0.004	0.961			− 0.073	0.327		
Hypertension	− 0.053	0.473			0.057	0.442			0.086	0.249		
DM	− 0.205	0.005	− 0.217	< 0.001	0.153	0.039	0.176	0.005	0.237	0.001	0.253	< 0.001
eGFR, mL/min/1.73m^2^	0.106	0.192			− 0.077	0.340			− 0.085	0.295		
NT-proBNP^a^	− 0.418	< 0.001	− 0.181	0.007	0.482	< 0.001	0.299	< 0.001	0.451	< 0.001	0.249	< 0.001
LGE type	− 0.424	< 0.001	− 0.379	< 0.001	0.384	< 0.001	0.280	< 0.001	0.393	< 0.001	0.320	< 0.001
LVMI, g/m^2^	− 0.381	< 0.001	− 0.269	< 0.001	0.348	< 0.001	0.209	0.001	0.305	< 0.001	0.177	0.007

*DM* diabetes mellitus, *RCM* restrictive cardiomyopathy, *BMI* body mass index, *BP* blood pressure, *NYHA* New York Heart Association, *HbA1c* glycated hemoglobin,; *eGFR* estimated glomerular filtration rate, *LVEDVi* left ventricular end diastolic volume index, *LVESVi* left ventricular end systolic volume index, *LVSVi* left ventricular stroke volume index, *LVEF* left ventricular ejection fraction, *LVMI* left ventricular mass index, *LVGFI* left ventricular global function index, *GPS* global peak strain, *PSSR* peak systolic strain rate, *PDSR* peak diastolic strain rate, *LGE* late gadolinium enhancement

^a^NT-proBNP was log-transformed before being included in the regression analysis

**Table 5:** Univariable and multivariable linear regression analysis of LV global peak strain rate in RCM Patients

	longitudinal PDSR			
	Univariable		Multivariable	
	r	p value	β	p value
Age#, years	− 0.051	0.491		
Male, n (%)	− 0.055	0.462		
BMI, kg/m^2^	0.122	0.101		
NYHA	− 0.252	0.001		
Hyperlipidemia	0.046	0.534		
Hypertension	0.092	0.216		
DM	− 0.269	< 0.001	− 0.263	< 0.001
eGFR, mL/min/1.73m^2^	0.091	0.264		
NT-proBNP^a^	− 0.363	< 0.001	− 0.196	0.008
LGE type	− 0.272	< 0.001	− 0.188	0.009
LVMI, g/m^2^	− 0.321	< 0.001	− 0.243	0.001

*DM* diabetes mellitus, *RCM* restrictive cardiomyopathy, *BMI* body mass index, *BP* blood pressure, *NYHA* New York Heart Association, *HbA1c* glycated hemoglobin,; *eGFR* estimated glomerular filtration rate, *LVEDVi* left ventricular end diastolic volume index, *LVESVi* left ventricular end systolic volume index, *LVSVi* left ventricular stroke volume index, *LVEF* left ventricular ejection fraction, *LVMI* left ventricular mass index, *LVGFI* left ventricular global function index, *GPS* global peak strain, *PSSR* peak systolic strain rate, *PDSR* peak diastolic strain rate, *LGE* late gadolinium enhancement, *PDSR* peak diastolic strain rate

^a^NT-proBNP was log-transformed before being included in the regression analysis

**Fig. 2:**
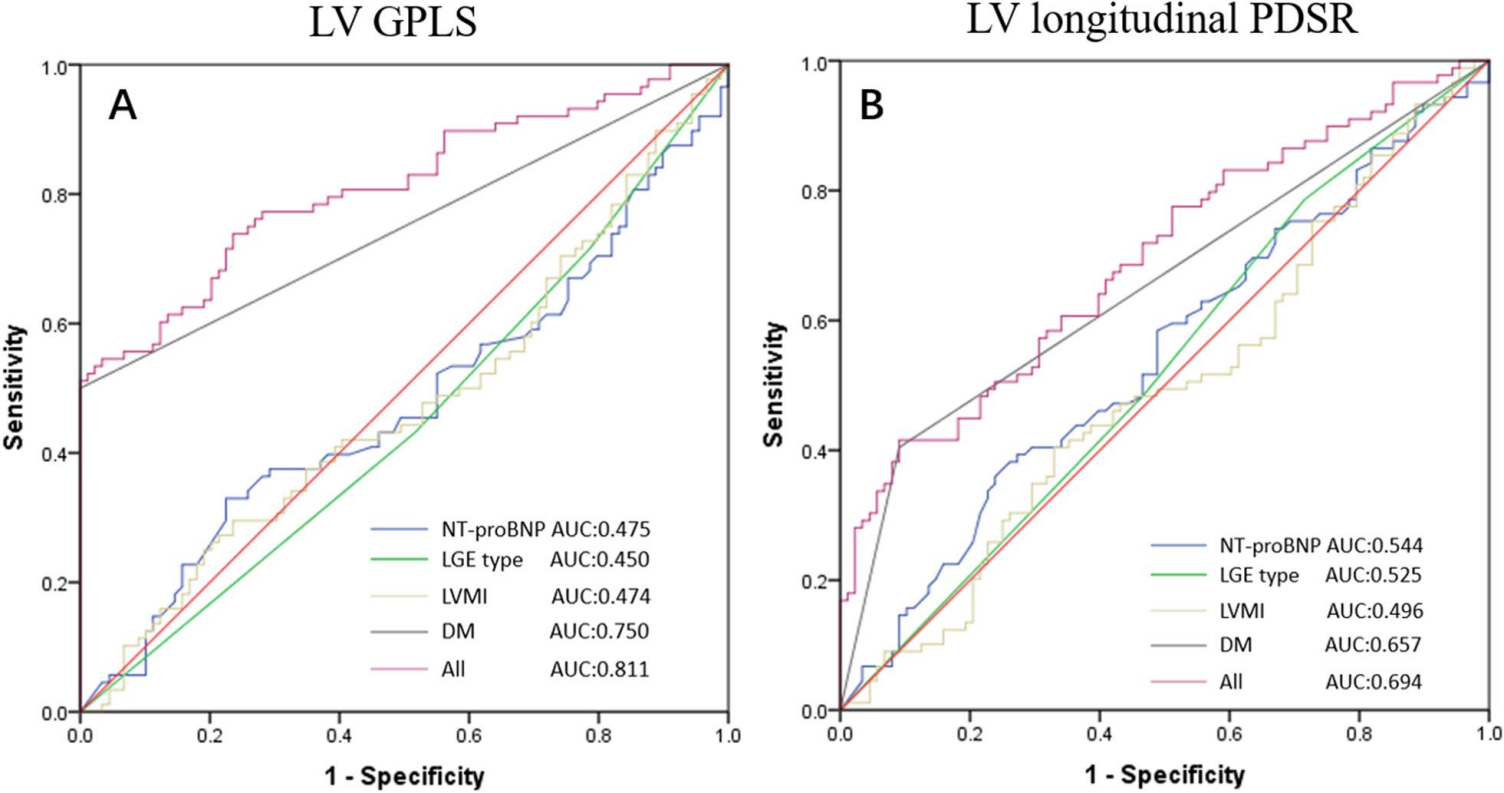
Receiver operating characteristic curve (ROC) analysis to predict the relationship with LV global longitudinal peak strain (**A**) and LV global longitudinal peak diastolic strain rate (**B**)

The results from the ROC analysis were showed in Fig. 2. The multiparameter combination, including DM, NT-proBNP, LGE type and LVMI showed a sensitivity of 54.6% and specificity of 96.6% to predict decreased LV GLPS (AUC = 0.81; 95% confidence interval = 0.75–0.87, p < 0.001), and a sensitivity of 41.6% and specificity of 90.9% to predict decreased LV longitudinal PDSR (AUC = 0.69; 95% confidence interval = 0.62–0.76, p < 0.001).

### Inter‑ and Intraobserver variability

There was excellent intra- and interobserver agreement in terms of LV global strain. The intra- and interobserver agreement was excellent for LV GPS (ICC = 0.923–0.978 and 0.912–0.961, respectively), LV global PSSR (ICC = 0.913–0.968 and 0.893–0.951, respectively), and LV global PDSR (ICC = 0.918–0.971 and 0.887–0.941, respectively) in all three directions.

## Discussion

This study investigated the difference in LV function and deformation damage in RCM patients with or without DM and explored the independent predictors of LV dysfunction and deformation injury. The main findings of this study are as follows: (1) For RCM patients, DM further deteriorated LV function, LV GPS in all three directions and LV peak diastolic strain rate in the longitudinal direction; (2) For RCM patients, DM was an independent determinant of impaired LVEF and LV GPS in all three directions; (3) Patients with RCM comorbid with DM displayed a decreased LV peak diastolic strain rate in the longitudinal direction, in which DM plays the predominant role. Our study demonstrated that DM may aggravate LV dysfunction and deformation injury in RCM patients, especially exacerbating the LV diastolic deformation rate injury in RCM patients. Therefore, RCM patients with comorbid DM may have a hidden high risk that needs more advanced and personalized management.

RCM is a heterogeneous group of heart muscle diseases characterized by restrictive ventricular physiology in the presence of normal or reduced diastolic volumes, with normal or near-normal LV systolic function and normal or near-normal wall thickness [7, 21]. Patients with RCM have an increased myocardial stiffness LV with impaired diastolic filling and high filling pressures. Chronically elevated LV diastolic pressures commonly induce pulmonary hypertension, right heart failure, and even whole heart failure [17]. As a cardiomyopathy with a poor prognosis, early intervention and control of risk factors for RCM can delay the further deterioration of cardiac function to a certain extent. As a growing health concern, DM is the most common chronic metabolic disease and the major risk factor for cardiovascular complications and adverse cardiovascular events [22]. Previous research has confirmed that diastolic dysfunction is an important damage stage in patients with DM, with the progression of the disease there are varying degrees of diffuse myocardial fibrosis [23–25]. We conducted this study to explore changes in LV dysfunction in RCM patients with DM. Our study demonstrated that conventional LV volume parameters (i.e., LVEDVi and LVESVi) were higher in RCM patients than the control group but similar between the two RCM groups. RCM patients with comorbid DM augmented the impaired LVSVi and LVGFI in RCM patients. LVGFI is a measure of LV cardiac performance that integrates LV structure into LV functional assessment, which can provide incremental prognostic value. Compared with LVEF, LVGFI mainly reflects structure-related LV function impairment. We speculated that DM may increase the stiffness of the LV, leading to a decrease in LVSVi and LVGFI without significant volume changes.

The underlying cause of cardiac alterations in RCM patients with DM is a combination of multiple mechanisms. Myocardial metabolism disorder is the characteristic of patients with DM, and the underlying mechanisms of how DM affects LV function may be due to the synthesis effect of metabolic disorders, excitation–contraction coupling impairment, microvasculature dysfunction, and extracellular matrix fibrosis [26, 27]. Several studies on DM-related myocardial damage have reported that DM can lead to more severe LV global deformation injury [13, 28]. Similarly, this study found that comorbid DM augmented the impairment of LV global peak strain in all three directions by CMR-FT in RCM patients. DM was an independent determinant of LV global peak strain, especially in the longitudinal direction in patients with RCM. The cardiac phenotypes of RCM are complex, with infiltrative cardiomyopathy such as cardiac amyloidosis being the most common type and infiltration starting in the sub-endocardium predominantly consisting of longitudinal fibers [29, 30]. Furthermore, the myocardial fiber in the sub-endocardium is the most susceptible to microvascular ischemia by DM [31, 32]. Therefore, the LV GLPS has a closed independent correlation with DM among the three directions. These pathomechanisms may partly explain the additive effect of DM on LV deformation in RCM patients.

In the early stages of RCM, LV diastolic dysfunction may occur due to increased myocardial stiffness, which causes a rapid rise in ventricular pressure at the beginning of the diastolic stage, while LV systolic function is typically preserved [7, 17]. In our study, the LV longitudinal PDSR was significantly decreased in the RCM(DM+) group, and multivariable regression analysis showed that DM was independently associated with longitudinal PDSR in RCM patients, which suggests a possible mechanistic link between DM and myocardial diastolic dysfunction in patients with RCM. Previous studies have shown that diastolic dysfunction can be detected in asymptomatic DM patients with normal LVEF levels, which is related to the complex mechanism of myocardium injury in diabetes [33, 34]. For patients with RCM, impaired diastolic dysfunction may be further aggravated with DM, which chronically elevates LV filling pressure and results in an almost fixed or decreased stroke volume. Under these conditions, the increase in heart rate is the only adaptive response to increased cardiac output, which is also consistent with the structure of our study.

Furthermore, our study showed that NT-proBNP levels were significantly higher in RCM(DM+) patients than in RCM(DM−) patients and were an independent determinant of LV global strains and longitudinal PDSR in RCM patients. LGE is associated with myocardial interstitial infiltration and is one of the most important CMR sign of RCM. Diffuse LGE independently predicted increased late mortality in RCM patients [20, 35]. Although there were no differences in the LGE type between RCM patients with and without DM, similar to NT-proBNP levels, the LGE type was also an independent determinant of LV deformation. The relationship of NT-proBNP and LGE type with LV deformation was stronger than that of DM. However, with the addition of DM, the multi-parameter combination obtained a larger AUC in the ROC curves of GLPS and longitudinal PDSR. In addition to the biochemical and imaging indicators of conventional cardiac function injury, DM, as a cardiovascular risk factor with increasing incidence, should be given more attention to achieve early prevention and treatment in RCM patients.

### Limitation

The study had several limitations. First, this was a retrospective single-center study, so there may be some selection bias in the results. The information on the onset, duration, and treatment of DM was unavailable for some patients due to the nature of the retrospective study. Second, not all patients received biopsies to confirm the cause of their restrictive cardiomyopathy, so we based the inclusion criteria on the clinical biopsy results or combined clinical, ECG, and imaging findings, according to the ESC review [6]. Third, RCM patients usually have several cardiovascular risk factors, including hypertension, hyperlipidemia and coronary heart disease, which may have potential adverse effects on LV function. In order to avoid ischemic myocardial damage caused by coronary heart disease, we excluded these patients. We included hypertension and hyperlipidemia in the multivariable regression analysis and found that DM was still an independent determinant of LV function.

## Conclusions

This study demonstrated that DM is an important risk factor for LV dysfunction and deformation injury in patients with RCM; DM may have an additive deleterious effect on LV dysfunction in patients with RCM, especially diastolic dysfunction in RCM patients. Early identification and initiation of treatment of DM in patients with RCM may improve prognosis.

## Data Availability

The datasets used and analyzed during the current study are available from the corresponding author on reasonable request.

## Abbreviations

RCM: Restrictive cardiomyopathy
DM: Diabetes mellitus
LV: Left ventricular
LVEF: Left ventricular ejection fraction
CMR: Cardiovascular Magnetic resonance imaging
LVEDVi: Left ventricular end diastolic volume index
LVESVi: Left ventricular end systolic volume index
LVSVi: Left ventricular stroke volume index
LVEF: Left ventricular ejection fraction
LVGFI: Left ventricular global function index
GRPS: Global radial peak strain
GCPS: Global circumferential peak strain
GLPS: Global longitudinal peak strain
BMI: Body mass index
HbA1c: Glycated hemoglobin
PSSR: Peak systolic strain rate
PDSR: Peak diastolic strain rate
